# A bibliometric analysis of acupuncture for cerebral infarction from 1993 to 2023

**DOI:** 10.3389/fneur.2024.1386164

**Published:** 2024-05-02

**Authors:** Yanqing Zhao, Li Huang, Wentao Li, Li Cai

**Affiliations:** Shanghai Municipal Hospital of Traditional Chinese Medicine, Shanghai University of Traditional Chinese Medicine, Shanghai, China

**Keywords:** acupuncture, cerebral infarction, VOSviewer, CiteSpace, bibliometric analysis

## Abstract

**Objective:**

This research aims to explore the trends and knowledge domain of acupuncture for cerebral infarction through bibliometrics.

**Methods:**

Publications related to acupuncture for cerebral infarction were retrieved from the Web of Science core collection database from 1993 to December 31, 2023. A domain knowledge graph was then constructed using VOSviewer, CiteSpace, GraphPad Prism, and Scimago Graphica.

**Results:**

The cumulative publication trend shows a steady increase over the years, with China being the most productive country. Notably, Europe exhibits significant close collaboration. Institutional cooperation is primarily observed among Chinese universities specializing in traditional Chinese medicine. Tao Jing is the most prolific author, with his highest number of publications is in “Stroke” journal, and Acupuncture Electro Therapeutics Research is the significant journal. Zhang SH is the most cited author, and Si QM is a prominent author in this field. Rehabilitation treatment after cerebral infarction emerges as a prevalent research focus, with nerve regeneration being a keyword. Long EZ's 1989 paper, published in the journal Stroke, holds significant importance. The prominent papers are Donnan et al. and Wu et al., which covers the following topics: “population-based study,” “Baihui Acupoint,” “memory deficits,” “neurotrophic factor,” and “randomized trial.”

**Conclusion:**

This bibliometric analysis of acupuncture for cerebral infarction offers insights into the Web of Science database, delineates a knowledge map of countries, authors, institutions, cited authors, keywords, cited references in the field of acupuncture for cerebral infarction, which has a momentous guiding significance for quickly and accurately positioning the key information in the field.

## 1 Introduction

Cerebral infarction, also known as ischemic stroke, is characterized by the localized necrosis of brain tissue caused by disruptions in cerebral blood circulation, leading to ischemia and hypoxia. It is associated with a high incidence rate, high disability rate, high mortality, and high recurrence rate (1–4). In China, the incidence rate of ischemic stroke revealed a slow upward trend, increasing from 129/100,000 in 2010 to 145/100,000 in 2019, with a prevalence rate of 1,256/100,000 in 2019 (5). The 1-year recurrence rate of stroke stands at 17% (6). The disability-adjusted life expectancy rose from 975/100,000 in 2005 to 1,007/100,000 in 2017. Vascular recanalization, encompassing intravenous thrombolysis and intravascular intervention, is considered the early effective treatment for cerebral infarction. However, the treatment time window is relatively short, and intravascular intervention expects doctors to have certain operational skills and surgical experience, which limits the widespread application of such treatment methods (7, 8). Modern rehabilitation technology has to some extent improved neurological dysfunction, but it has yet to address the challenges of high stroke recurrence and disability rates (9). Traditional Chinese medicine offers a complementary approach by enhancing neurological function recovery and quality of life through syndrome-specific interventions. Combining of traditional Chinese medicine with Western medicine has synergistic effects in managing cerebral infarction across various stages, supported by numerous clinical studies and basic research, confirming its efficacy and safety. Acupuncture, as a non-pharmacologic intervention within traditional Chinese medicine, holds promise for the treatment of stroke (10).

Bibliometrics is an interdisciplinary field that employs mathematical and statistical methods to quantitatively analyze various forms of knowledge dissemination (11). Through the examination of countries, institutions, authors, keywords, and references, we can discern the research hotspots, research frontiers, and research content within the given field. Software tools, such as Citespace (12), VOSviewer (13), and Histcite (14), facilitate bibliometric analysis. Previous bibliometrics studies on acupuncture in cerebral infarction treatment mainly include “Visual bibliometric analysis of electroacupuncture research in stroke treatment: a 20-year overview” by Chun et al. (15) and “Global trends and development of acupuncture for stroke: A review and bibliometric analysis” by Cheng and Yu (16). Hyonjun Chun's research focused only on electroacupuncture, whereas Cheng CJ's expanded his search strategy to moxibustion. Our study specifically delves into the bibliometric patterns of acupuncture in treating ischemic stroke, aiming to propose future research directions in this domain. Combining CiteSpace with VOSviewer, we construct a knowledge graph encompassing countries, institutions, journals, authors, cited authors, keywords, and cited references. We employ burst detection, cluster analysis, and Rainbow maps to identify hotspots and trends within the field.

## 2 Methods

### 2.1 Source of literature

First, we collected the synonyms for “cerebral infarction” and “acupuncture” through the MeSH Database in PubMed and then compiled the final data. Next, we imported the Web of Science database with English Topic = “cerebral infarction,” chose “AND” as the logical relation word, and retrieved the literature literally. The search plan is as follows: TS = “cerebral infarction” AND “acupuncture;” TS = “cerebral infarction” AND “acupuncture therapy;” TS = “cerebral infarction” AND “Acupuncture, Ear;” TS = “cerebral infarction” AND “Acupuncture Points;” TS = “cerebral infarction” AND “Acupuncture Analgesia” separately (17). We will use the topic word “ischemic stroke” along with synonyms for “acupuncture” to install the above strategy and then search the database. Then, for the parameter settings in the WoS database, we selected “Science Citation Index Expand” and “Social Sciences Citation Index” for the “Citation Index” option. The search date in the WoS database is January 1, 2024, and the “Add Date Range” is set from January 1, 1993, to December 31, 2023. The search strategy is shown in Supplementary Table S1. The language of the literature is not limited. After retrieving the literature, we merged the obtained data and then manipulated the data using the Citespace software to conduct deduplication operations. To further ensure the accuracy of the data, we also adopted a double-person comparison input data method to perform deduplication to ensure its uniqueness, resulting in 448 pieces of literature. It is noteworthy that the Web of Science database originates from the Peking University Library database in China.

### 2.2 Data analysis

Literatures exported from the Web of Science database were imported into CiteSpace 5.1.R8.SE and VOSviewer 1.6.19. CiteSpace 5.1.R8.SE was used for implementing clustering analysis of cited journals, authors, cited authors, keywords and cited references, and journal co-citation network, detection of burst keywords, cited authors, and cited references. VOSviewer 1.6.19 was used to fulfill network visualization analysis of countries and organizations, author and cited author, density visualization of organizations, authors, cited authors, and cited references. GraphPad Prism 8 is used to construct trend charts for the total number of publications and the cumulative total number of publications. Scimago Graphica was used to visualize the global distribution of publications. Moreover, CiteSpace extends the module value (named Q value) and the silhouette value (named S value) to estimate the impression of the atlas depicted. The Q value is generally in the interval [0,1], and Q > 0.3 implies that the allocated community structure is conspicuous. When S > 0.7, the clustering is the most reliable; if S > 0.5, clustering is generally moderate (18).

In the density map of clustering analysis using VOSviewer software, the higher a rainbow image is referenced, the darker the color (19). The size of nodes in the network map of countries, institutions, authors, and cited authors represents the number of published papers, the size of nodes in the keyword node network diagram represents the frequency of occurrence, and the size of nodes in the reference network diagram indicates the number of common citations among each other. The parameter settings in Citespace software are as follows: #Years Per Slice is set to 3, Pruning is set to “Pathfinder,” “Pruning Sliced networks,” and “Pruning the merged network.” **Pathfinder:** Refers to pruning the research network using path lookup algorithms. The path search algorithm starts from the starting point, gradually searches for all other nodes, and then prunes the network based on the importance of the nodes. **Pruning Sliced Networks** refers to pruning a sharded network, retaining only nodes and edges related to the main network and filtering out other nodes and edges. **Pruning the Merged Network** refers to pruning the merged network, retaining only nodes and edges related to the research problem, filtering out other nodes and edges, and making the structure of the research network simpler and clearer. The cluster analysis results mainly include cluster ID, mean year, size, silhouette, label (LLR), and label (MI). Cluster ID is the number obtained after clustering, and size represents the number of members contained in the cluster. The larger the size, the smaller the number. Mean year represents the average year of the literature in the cluster, which can be used to judge the distance of the cited literature in the cluster. The larger the log-likelihood ratio (LLR), the more representative the cluster category; mutual information (MI) is mainly used to represent the relationship between terms and categories in text mining, and it does not consider the frequency of feature words.

## 3 Results

### 3.1 Analysis of the total number of publications and accumulated total number of publications

**Figure 1:**
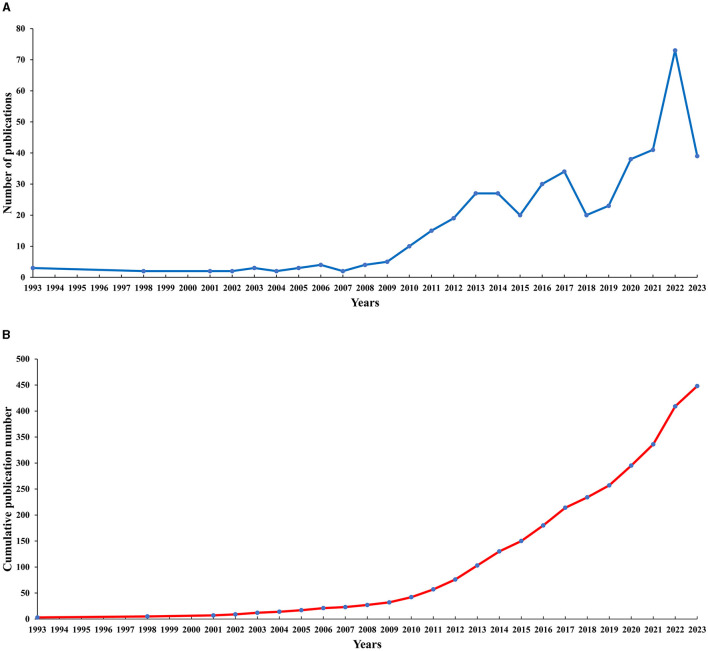
The number of publications and Cumulative publication number on acupuncture for cerebral infarction from 1993 to 2023.

The earliest research in this study can be traced back to 1993 where only three articles were published, and it reached 73 in 2022 and 39 in 2023. From 1993 to 2007, the output of publications remained stable at a baseline level of growth, but the annual growth was less, and even no relevant research was published from 1994 to 1997, 1999, or 2000 (see Figure 1A). The average total number of publications fluctuated slightly around 2.5. From 2008 to 2018, the total number of publications showed an increasing trend year by year, with an average output, which was stable at around 19, showing a significant trend compared to 1993 to 2007. However, there were some fluctuations between 2015 and 2018. In 2018–2023, the growth trend was very obvious, with an average total number of publications reaching 39 (see Figure 1B) and the overall trend of the cumulative total number of publications rose year by year.

### 3.2 Analysis of countries and institutions

**Figure 2:**
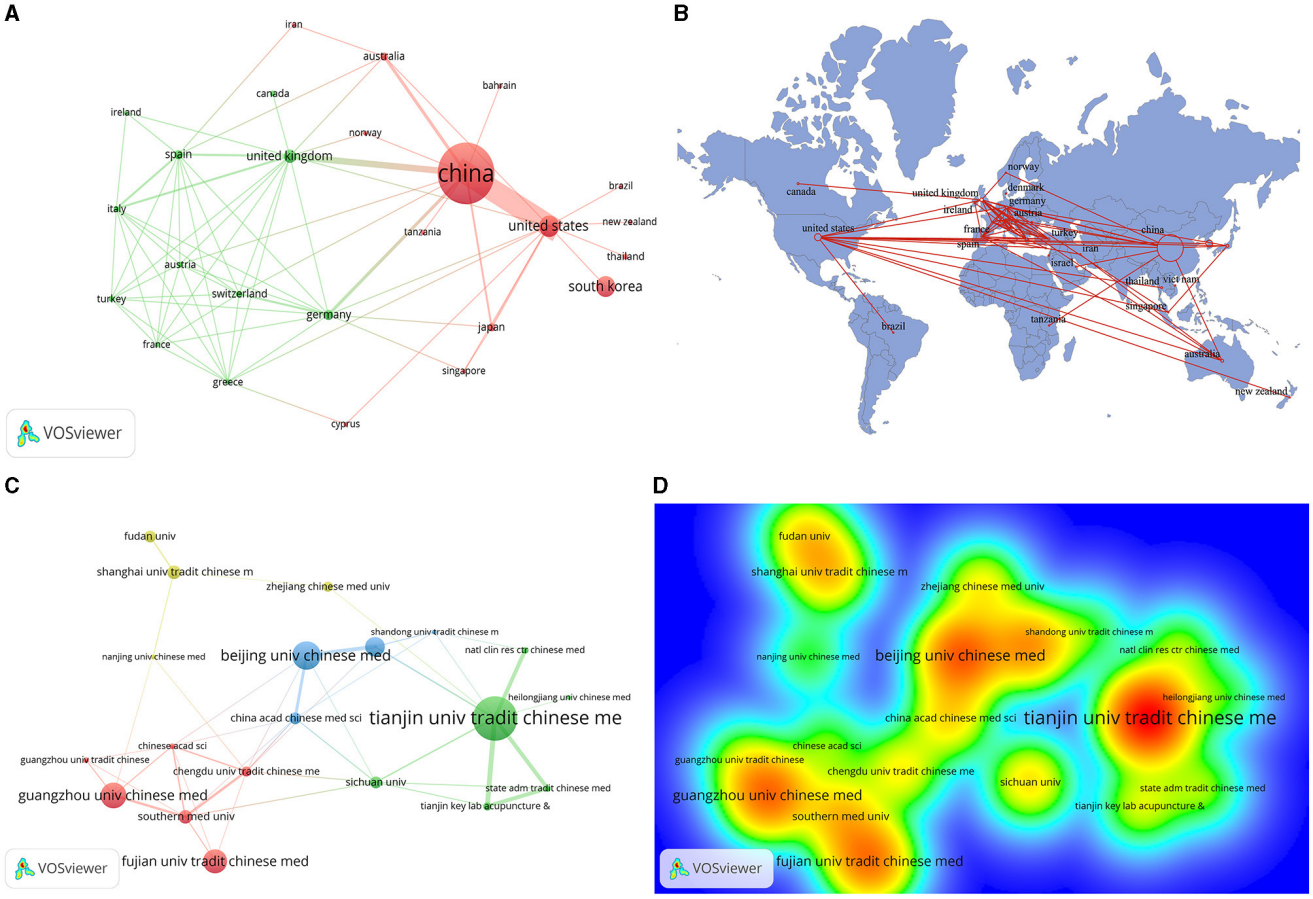
The visualization of countries **(A)**, geographical distribution **(B)**, institutions **(C)**, and Rainbow map on acupuncture for cerebral infarction from 1993 to 2023. (**A**: different nodes represent different countries, and the size of the nodes represents the number of publications; **B**: the color shades represent the number of published publications; **C**: different nodes represent different institutions, and the size of the nodes represents the number of publications; **D**: the darker the yellow, the higher the frequency of appearance).

The research involves 30 countries and 540 organizations, among which China, USA, South Korea, United Kingdom, and Germany are the top 5 countries, and the most cited countries are China (citation = 5,066) and South Korea (citation = 726) (Table 1). Figures 2A, B reveal that the strongest cooperative relationships are between China and the USA, followed by China and the United Kingdom. From the perspective of the cooperative relationship network, it is evident that the relationship is active among European countries.

**Table 1:** Countries and organizations contributed to publications on acupuncture for cerebral infarction from 1993 to 2023.

**Country**	**Documents**	**Citations**	**Organization**	**Documents**	**Citations**
China	377	5,066	Tianjin univ tradit Chinese med	52	398
USA	34	698	Beijing University Chinese med	33	315
South Korea	33	726	Guangzhou Univ Chinese Med	30	217
United Kingdom	12	365	Fujian Univ Tradit Chinese Med	28	820
Germany	7	88	Capital Med Univ	23	466
Spain	5	83	China Med Univ	21	492
Australia	4	61	China med univ Hosp	19	433
Japan	4	46	Kyung Hee Univ	18	300
Switzerland	3	53	Shanghai Univ Tradit Chinese Med	16	60
Austria	2	62	Southern Med Univ	16	152
Canada	2	84	China Acad Chinese Med Sci	14	101
Greece	2	52	Fudan Univ	14	155
Italy	2	48	Sichuan Univ	14	459
Thailand	2	20	Chengdu Univ Tradit Chinese Med	12	134
Turkey	2	44	Zhejiang Chinese Med Univ	12	132

In this table, the other two identical columns represent different rankings. The left column of the table represents the Countries that have published acupuncture for cerebral infarction. The frequency of contribution is sorted from high to low. Guangzhou University Chinese med, Guangzhou University of Chinese Medicine; Fujian univ tradit Chinese med, Fujian University of Traditional Chinese Medicine; Capital Med univ, Capital Medical University; china acad Chinese Med sci, China Academy of Chinese Medical Sciences; Tianjin univ tradit Chinese med, Tianjin University of Traditional Chinese Medicine; Beijing univ chinese med, Beijing University of Chinese Medicine; china med univ, China Medical University; china med univ hosp, China Medical University Hospital; kyung hee univ, kyung hee university; shanghai univ tradit Chinese med, Shanghai University of Traditional Chinese Medicine; southern med univ, Southern medical university; Fudan univ, Fudan University; Sichuan univ, Sichuan University; Chengdu univ tradit Chinese med, Chengdu University of Traditional Chinese Medicine; Zhejiang Chinese med univ, Zhejiang Chinese Medical University.

The top five institutions ranked by output (Table 1) are Tianjin University of Traditional Chinese Medicine, Beijing University of Chinese Medicine, Guangzhou University of Chinese Medicine, Fujian University of Traditional Chinese Medicine, and Capital Medical University, while Fujian University of Traditional Chinese Medicine (citation = 820) has the most cited output. In addition to verifying the aforementioned rules, Figures 2C, D also indicate that the cooperative relationships of the institutions mainly include four parts: a cooperative network centered around Beijing, the capital of China; a collaborative network converged around Tianjin; the southern cooperative relationship with Guangzhou as the core; a cooperative relationship network concentrated on the Yangtze River Delta. The active relationship networks of these organizations come from China. Furthermore, these cooperative relationships are only curtailed to local organizations in first-tier cities in China and lack cooperation with other national institutions.

### 3.3 Analysis of authors

**Figure 3:**
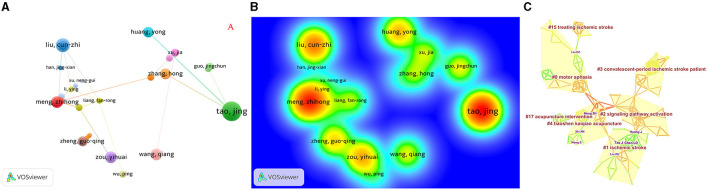
The visualization of authors **(A)**, Rainbow map **(B)**, and Cluster map **(C)** of authors based on label clusters with title terms. (**A**: different nodes represent different authors, and the size of the nodes represents the number of publications; **B**: the darker the yellow, the higher the frequency of appearance; **C**: different color blocks represent different clustering categories).

Analyzing the volume of published literature and citations, Table 2 presents the top 10 authors contributing articles to this field. Tao Jing, with a frequency of 21 publications, was posited as the most active author in the field, followed by Chen Lidian, Huang Jia, Liu Cun-zhi, and Liu Weilin (Figures 3A, B). Moreover, Tao Jing is the most cited author and the most prominent researcher.

**Table 2:** Authors, cited authors, keywords and cited references contributed to publications on acupuncture for cerebral infarction from 1993 to 2023.

**No**.	**Author**	**Frequency**	**Citations**	**Cited Author**	**Citations**	**Total link strength**	**Cited Reference**	**Frequency**	**Cited Reference**	**Centrality**	**Keyword**	**Frequency**
1	Tao, Jing	21	710	Zhang, SH	75	705	Wu et al. (20)	41	Wang et al. (21)	0.58	Acupuncture	319
2	Chen, Lidian	20	668	Longa, EZ	74	334	Chavez et al. (22)	40	Lin and Hsieh (23)	0.54	Stroke	167
3	Huang, Jia	14	550	Wu, P	73	671	Zhang et al. (24)	36	Lu et al. (25)	0.52	Ischemic Stroke	122
4	Liu, Cun-Zhi	12	375	Wang, Q	69	615	Yang et al. (26)	30	Kim et al. (27)	0.51	Recovery	69
5	Liu, Weilin	12	292	Wang, Y	66	619	Wang et al. (28)	27	Xie et al. (29)	0.50	Rehabilitation	56
6	Meng, Zhihong	9	31	Zhang, Y	54	591	Kong et al. (30)	27	Kim et al. (31)	0.48	Brain	53
7	Wang, Qiang	9	278	Tao, J	51	561	Shen et al. (32)	25	Liu et al. (33)	0.38	Expression	49
8	Yang, Shanli	9	334	Li, J	49	521	Lu et al. (25)	24	Li et al. (19)	0.29	Stimulation	49
9	Zou, Yihuai	9	80	Zhou, F	48	595	Kim et al. (27)	22	Benjamin et al. (34)	0.25	Activation	42
10	Huang, Yong	8	108	Feigin, VL	46	317	Donnan et al. (35)	20	Zhao et al. (36)	0.24	Rats	33

In this table, the other two identical columns represent different rankings. The left column of the table represents the authors who have published acupuncture for cerebral infarction. The frequency of contribution is sorted from high to low.

Through a refined approach using Citespace software, the process involved selecting the pathfinder, pruning sliced networks, and pruning the merged network. Years per slice were set to 3 years in the clipping mode, and using the cosine algorithm yeilded clustering analysis results (Figure 3C). A total of 17 clustering words were obtained (Table 3): #0 motor aphasia, #1ischemic stroke, #2 signaling pathway activation, #3 convalescent-period ischemic stroke patient, #4 tiaoshen kaiqiao acupuncture, #15 treating ischemic stroke, and #17 acupuncture intervention. Thus, it can be seen that scholars often conduct related research on the sequelae of cerebral infarction and recovery period ischemic stroke using acupuncture treatment methods.

**Table 3:** Authors engaged in acupuncture for cerebral infarction that details of knowledge clusters.

**Cluster ID**	**Size**	**Silhouette**	**Mean (Year)**	**Label (LLR)**	**Label (MI)**
0	23	0.911	2017	Motor aphasia	Prolonged flaccid paralysis
1	23	0.965	2014	Ischemic stroke	Prolonged flaccid paralysis
2	19	0.849	2021	Signaling pathway activation	Prolonged flaccid paralysis
3	18	0.971	2018	Convalescent-period ischemic stroke patient	Prolonged flaccid paralysis
4	17	0.869	2016	Tiaoshen kaiqiao acupuncture	Prolonged flaccid paralysis
15	6	0.992	2018	Treating ischemic stroke	Motor aphasia
17	5	0.979	2014	Acupuncture intervention	Motor aphasia

The cluster analysis results mainly include cluster ID, mean year, size, silhouette, label (LLR), and label (MI). Cluster ID is the number after clustering, and Size represents the number of members contained in the cluster. The larger the size is, the smaller the number. Mean Year represents the average year of the literature in the cluster, which can be used to judge the distance of the cited literature in the cluster. The larger the log-likelihood ratio (LLR) is, the more representative the cluster category; mutual information (MI) is mainly used to represent the relationship between terms and categories in text mining, and it does not consider the frequency of feature words.

### 3.4 Analysis of cited authors

**Figure 4:**
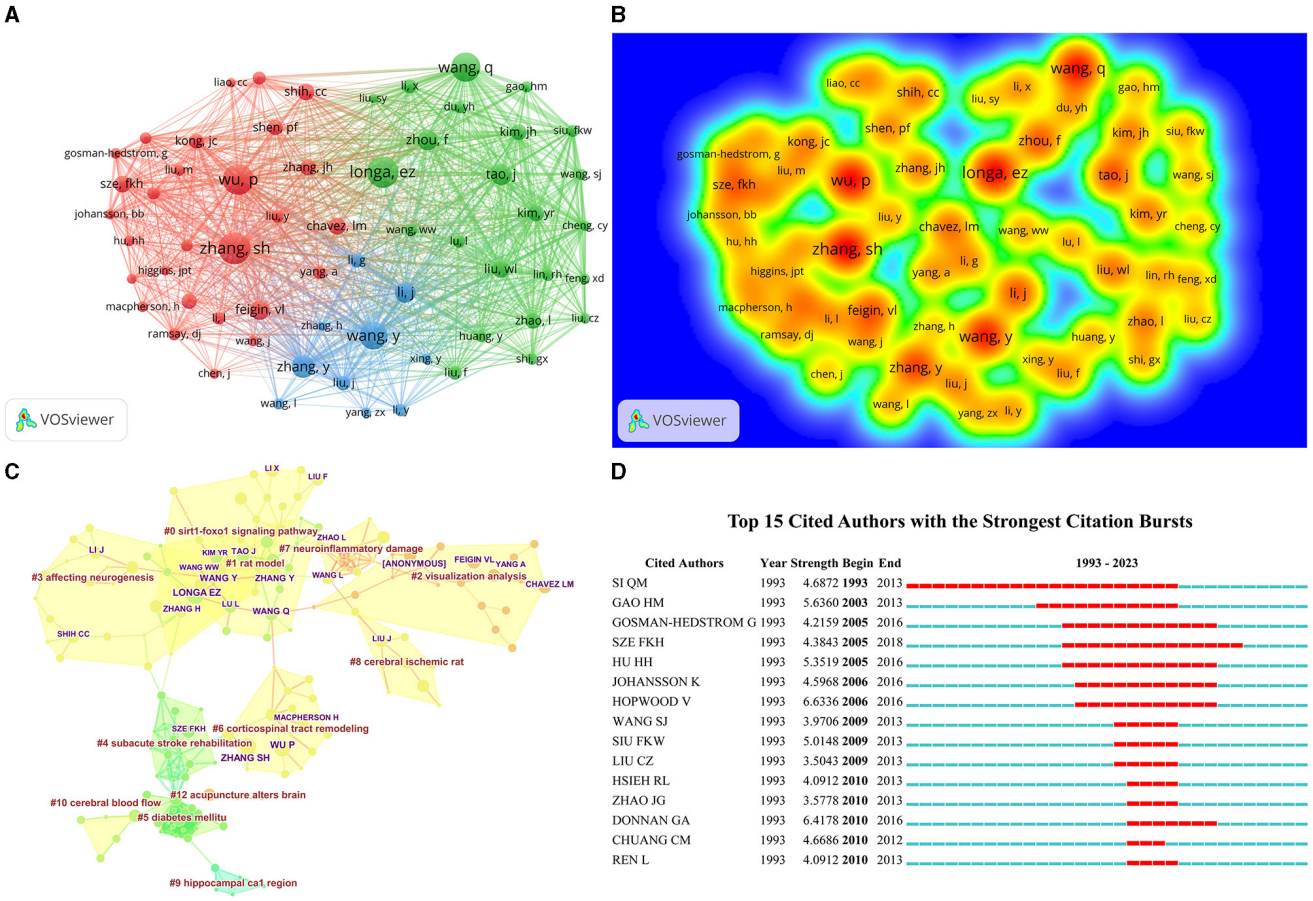
The visualization of cited authors **(A)**, Rainbow map **(B)**, Cluster map **(C)**, and Citation bursts **(D)** of cited authors based on label clusters with title terms. (**A**: different nodes represent different cited authors, and the size of the nodes represents the number of publications; **B**: the darker the yellow, the higher the frequency of appearance; **C**: different color blocks represent different clustering categories; **D**: the red bar represents frequent citations, while the green bar represents infrequent citations).

Cited authorship refers to a phenomenon in which two authors are jointly cited by other literature. When Citespace software is used to calculate the co-citation of authors, only the co-citation of the first author is considered, and even if the same author is cited multiple times in the same literature, it is counted as one citation. By calculating the co-citation author relationship, a co-citation network diagram of authors can be achieved, which can proclaim the academic community of a certain research field. Among all co-cited authors, 7 authors have been co-cited more than 50 times (Table 2). The author with the most co-citations is Zhang SH (citation = 75), followed by Longa, EZ (citation = 74), and Wu P (citation = 73). In addition, 126 authors with more than 15 co-citations were filtered to draw a co-citation network diagram (Figures 4A, B). Active collaboration between different co-cited authors was also observed, for example, Tao J collaborated with Wu P, Zhang SH, and Longa EZ. By rerunning the Citespace software, we acquired clustering analysis results (Figure 4C), and a total of 12 clustering words were obtained (Table 4): #0 Sirt1-foxo1signaling pathway, #1 rat model, #2 visualization analysis, #3 affecting neurogenesis, #4 subacute stroke rehabilitation, #5 diabetes mellitus, #6 corticospinal tract remodeling, #7 neuroinflammatory damage, #8 cerebral ischemic rat, #9 hippocampal ca1 region, #10 cerebral blood flow, and #12 acupuncture alters the brain. The strongest citation burst detection is used to report research hotspots within a certain time node. By utilizing the “Citation/Frequency Burst History” function in the “Visualization” section of Citespace software, the strongest citation burst detection results can be gained. The red bar represents the duration of the citation burst and the start and end years of the citation burst (11), which prompts that Si QM is the hot topic author in this fields (Figure 4D).

**Table 4:** Cited authors engaged in acupuncture for cerebral infarction that details of knowledge clusters.

**Cluster ID**	**Size**	**Silhouette**	**Mean (Year)**	**Label (LLR)**	**Label (MI)**
0	25	0.890	2014	Sirt1-foxo1signaling pathway	Electro-acupuncture treatment
1	20	0.908	2013	Rat model	Electro-acupuncture treatment
2	20	0.892	2020	Visualization analysis	Electro-acupuncture treatment
3	20	0.847	2015	Affecting neurogenesis	Electro-acupuncture treatment
4	18	0.924	2005	Subacute stroke rehabilitation	Subacute stroke rehabilitation
5	18	0.967	2009	Diabetes mellitu	Electro-acupuncture treatment
6	16	0.939	2016	Corticospinal tract remodeling	Electro-acupuncture treatment
7	11	0.966	2019	Neuroinflammatory damage	Electro-acupuncture treatment
8	9	0.926	2018	Cerebral ischemic rat	Electro-acupuncture treatment
9	5	0.980	2003	Hippocampal ca1 region	Ischemic stroke
10	5	0.973	2011	Cerebral blood flow	Ischemic stroke
12	3	0.937	2020	Acupuncture alters brain	Ischemic stroke

The cluster analysis results mainly include cluster ID, mean year, size, silhouette, label (LLR), and label (MI). Cluster ID is the number after clustering, and Size represents the number of members contained in the cluster. The larger the size is, the smaller the number. Mean Year represents the average year of the literature in the cluster, which can be used to judge the distance of the cited literature in the cluster. The larger the log-likelihood ratio (LLR) is, the more representative the cluster category; mutual information (MI) is mainly used to represent the relationship between terms and categories in text mining, and it does not consider the frequency of feature words.

### 3.5 Analysis of journals

The distribution of the elemental journals within the acupuncture research pertaining to cerebral infarction is shown in Table 5. The most prolific journal is Stroke (Frequency = 349), followed by Evidence-based Complementary and Alternative Medicine (Frequency = 192), Plos One (Frequency = 175), Journal of Alternative and Complementary Medicine (Frequency = 147), and Lancet (Frequency = 144). The most critical journal is Acupuncture Electro Therapeutics Research (centrality = 0.75), followed by Brain Research (centrality = 0.63), Journal of Traditional Chinese Medicine (centrality = 0.56), Stroke (centrality = 0.51), and Journal of Cerebral Blood Flow And Metabolism (centrality = 0.39). The average impact factor of the top 10 journals in this research field is 21.155. Half of the journals have an IF>5 and are recognized as the most notable journals in the Journal co-citation network relationship. Moreover, it can be seen that acupuncture is increasingly recognized.

**Table 5:** Journals contributed to publications on acupuncture for cerebral infarction from 1993 to 2023.

**Journal**	**Frequency**	**IF (2022)**	**Journal**	**Centrality**	**IF (2022)**
Stroke	349	8.40	Acupuncture Electro Therapeutics Research	0.75	0.30
Evidence-based Complementary and Alternative Medicine	192	2.65	Brain Research	0.63	2.90
PLoS ONE	175	3.70	Journal of Traditional Chinese Medicine	0.56	2.60
Journal of Alternative and Complementary Medicine	147	2.60	Stroke	0.51	8.40
LLancet	144	168.90	Journal of Cerebral Blood Flow And Metabolism	0.39	6.30
Journal of Traditional Chinese Medicine	144	2.60	Neuroscience Letters	0.29	2.50
American Journal of Chinese Medicine	141	**5.70**	Journal of Neurolinguistics	0.29	2.00
Neuroscience Letters	139	2.50	Evidence-based Complementary and Alternative Medicine	0.28	2.65
Cochrane Database of Systematic Reviews	138	**8.40**	Clinical Rehabilitation	0.28	3.00
Neural Regeneration Research	134	6.10	Journal of Alternative and Complementary Medicine	0.27	2.60

In this table, the other two identical columns represent different rankings. The left column of the table represents the journals that have published acupuncture for cerebral infarction. The frequency of contribution is sorted from high to low. The column on the right represents the sorting from high to low according to centrality. The bold values represent the size of the impact factor for the journal in 2022. For example, 5.7 represents the impact factor for “American Journal of Chinese Medicine” in 2022, while the bold number 8.40 represents the size of the impact factor for “Cochrane Database of Systematic Reviews” in 2022.

**Figure 5:**
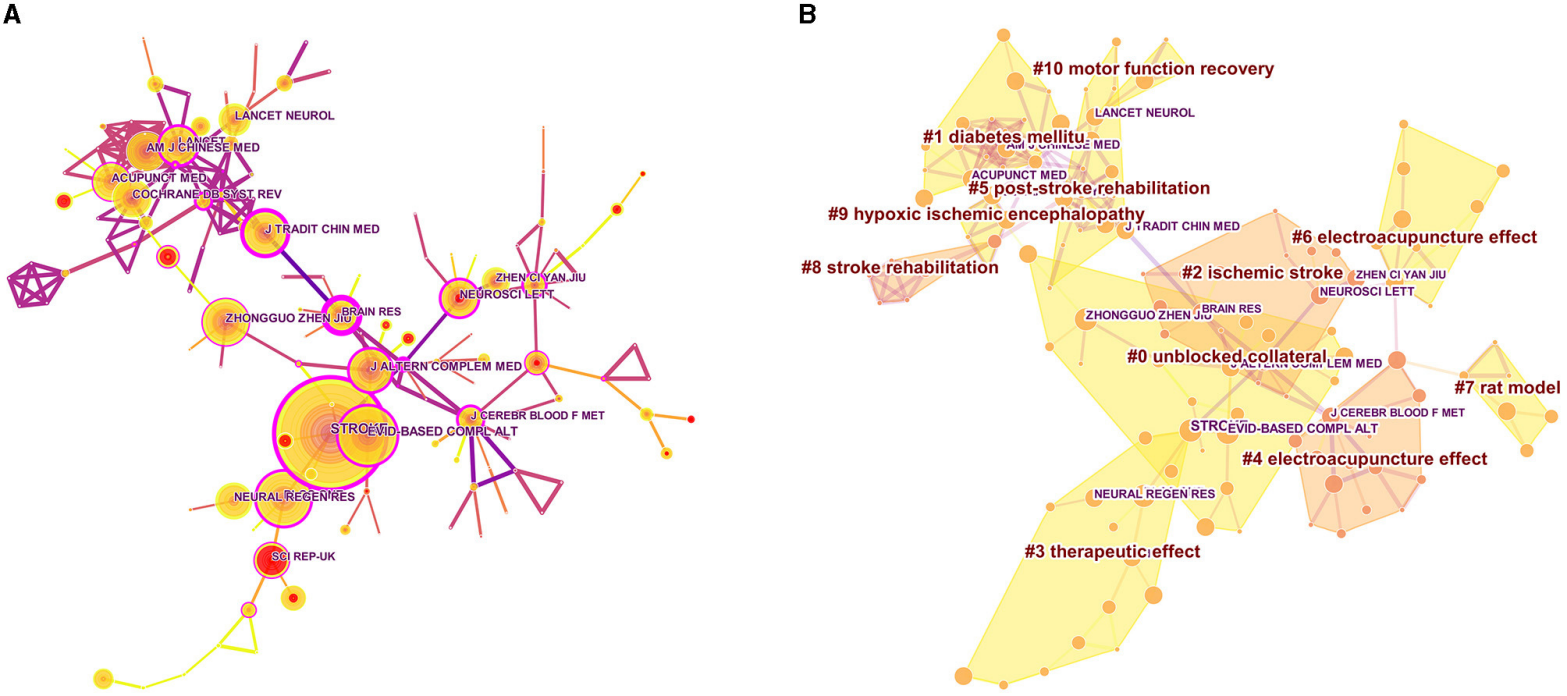
Network of journals **(A)** and Cluster map **(B)** of authors based on label clusters with title terms on acupuncture for cerebral infarction. **(A)** The purple node in the middle of the annual ring means the influence and the significance of a journal. The larger the node and the more purple it exhibits, the greater is the importance of the journal. **(B)** Different color blocks represent different clustering categories.

Eleven cluster atlases with 152 nodes and 215 links (Figure 5A, Modularity Q = 0.7764, Silhouette = 0.4098) of journals were manifested by Citespace software, along with a timeline view (Figure 5B). The modularity Q > 0.7 hints that the results of the divided community structure are infallible. Then, the researcher can quickly fix the position of the analogous research content and the relationship between dissimilar journals. The results of the cluster analysis are shown in Table 6: #0 unblocked collateral, #1 diabetes mellitus, #2 ischemic stroke, #3 therapeutic effect, #4 electroacupuncture effect, #5 post-stroke rehabilitation, #6 electroacupuncture effect, #7 rat model, #8 stroke rehabilitation, #9 hypoxic-ischemic encephalopathy, and #10 motor function recovery.

**Table 6:** Cited journal engaged in acupuncture for cerebral infarction that details knowledge clusters.

**Cluster ID**	**Size**	**Silhouette**	**Mean (Year)**	**Label (LLR)**	**Label (MI)**
0	20	0.911	2012	Unblocked collateral	Following acute middle cerebral artery infarction
1	19	0.907	2010	Diabetes mellitu	Global ischemia model
2	15	0.909	2009	Ischemic stroke	Following acute middle cerebral artery infarction
3	15	0.963	2014	Therapeutic effect	Following acute middle cerebral artery infarction
4	15	0.900	2009	Electroacupuncture effect	Following acute middle cerebral artery infarction
5	14	0.907	2006	Post-stroke rehabilitation	Subacute stroke rehabilitation
6	11	0.987	2012	Electroacupuncture effect	Post-stroke spasticity rat
7	6	0.983	2012	Rat model	Following acute middle cerebral artery infarction
8	6	0.929	2005	Stroke rehabilitation	Ischemic stroke
9	5	0.969	2005	Hypoxic ischemic encephalopathy	Ischemic stroke
10	4	0.929	2006	Motor function recovery	Ischemic stroke

The cluster analysis results mainly include cluster ID, mean year, size, silhouette, label (LLR), and label (MI). Cluster ID is the number after clustering, and Size represents the number of members contained in the cluster. The larger the size is, the smaller the number. Mean Year represents the average year of the literature in the cluster, which can be used to judge the distance of the cited literature in the cluster. The larger the log-likelihood ratio (LLR) is, the more representative the cluster category; mutual information (MI) is mainly used to represent the relationship between terms and categories in text mining, and it does not consider the frequency of feature words.

### 3.6 Analysis of keywords

**Figure 6:**
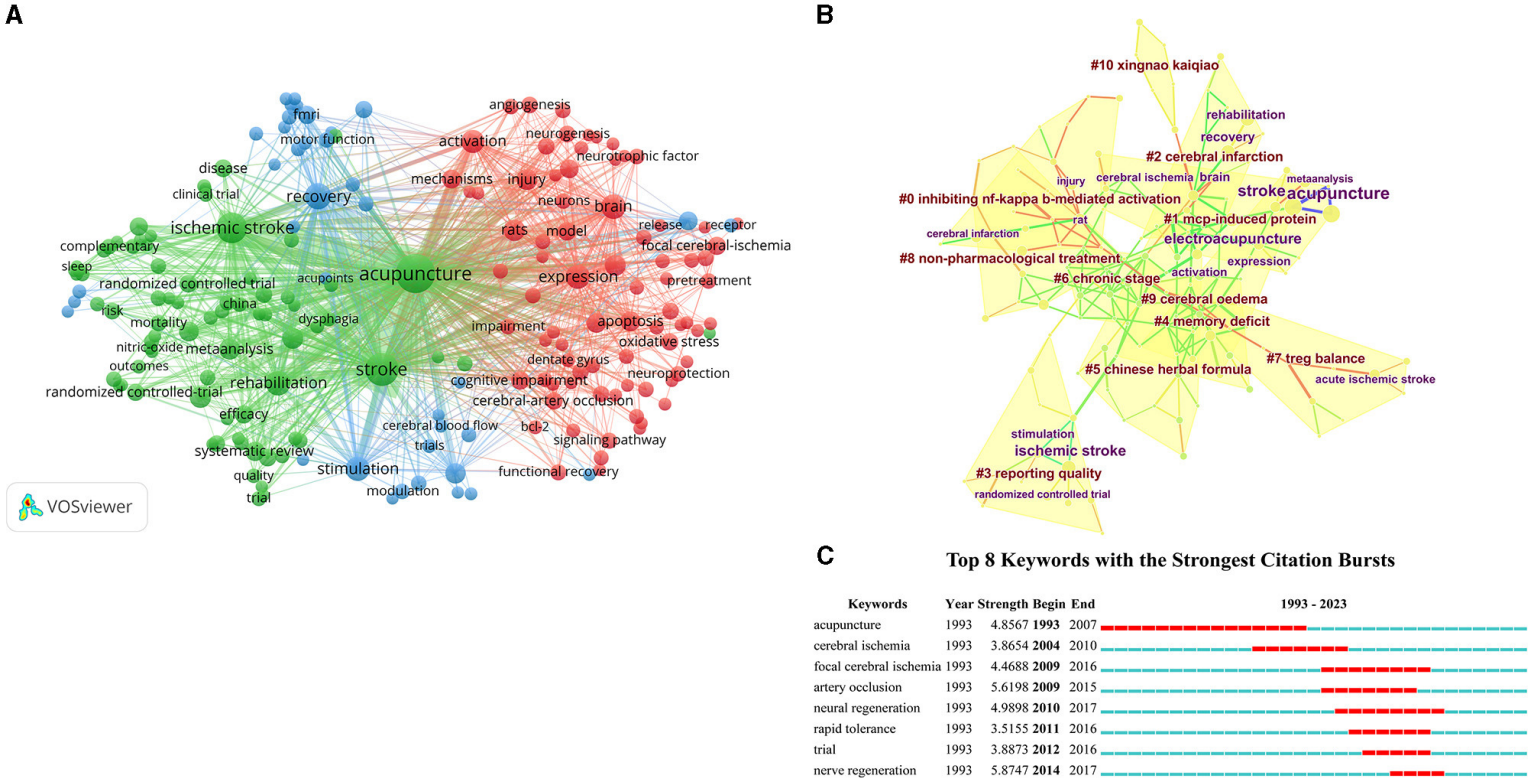
The visualization of keywords **(A)**, Cluster map **(B)**, and Citation bursts **(C)** of keywords based on label clusters with title terms. (**A**: different nodes represent different keywords, and the size of the nodes represents the number of frequencies; **B**: different color blocks represent different clustering categories; **C**: the red bar represents frequent citations, while the green bar represents infrequent citations).

By conducting a co-occurrence analysis of keywords, we can pinpoint the prevailing themes within the domain of acupuncture for cerebral infarction. The frequency of the top 10 keywords in this research field exceeds 30, representing the main direction of acupuncture for cerebral infarction (Table 2, Figure 6A). Setting aside the two research topics of acupuncture and cerebral infarction, we detected that research in this field is more concentrated on the sequelae of cerebral infarction during the recovery period.

Eleven clusters were obtained, with a modularity Q of 0.769. The mean Silhouette was 0.5914 > 0.3. The 11 largest clusters are identified in Table 7. The cluster view bespeaks the co-occurring author keywords and keywords plus (Figure 6B), comprised #0 inhibiting nf-kappa-b-mediated activation, #1 MCP-induced protein, #2 cerebral infarction, #3 reporting quality, #4 memory deficit, #5 Chinese herbal formula, #6 chronic stage, #7 treg balance, #8 non-pharmacological treatment, #9 cerebral oedema, and #10 Xingnao Kaiqiao. Next, by analyzing the Citation/Frequency Burst History, we will identify the hotspots (Figure 6C) of the keywords. Putting aside the two main themes of acupuncture and cerebral infarction, we discovered that the research hotspots in this field focus more on nerve regeneration.

**Table 7:** Keywords related to acupuncture for cerebral infarction that details knowledge clusters.

**Cluster ID**	**Size**	**Silhouette**	**Mean (Year)**	**Label (LLR)**	**Label (MI)**
0	18	0.938	2015	Inhibiting nf-kappa-b-mediated activation	Local anesthesia
1	16	0.798	2008	Mcp-induced protein	Local anesthesia
2	15	0.855	2012	Cerebral infarction	Local anesthesia
3	14	0.834	2014	Reporting quality	Local anesthesia
4	14	0.872	2012	Memory deficit	Local anesthesia
5	14	0.889	2013	Chinese herbal formula	Acupuncture therapy
6	14	0.712	2012	Chronic stage	Brain disease
7	13	0.934	2018	Treg balance	Local anesthesia
8	10	0.844	2015	Non-pharmacological treatment	Unblocked collateral
9	8	0.813	2011	Cerebral oedema	Unblocked collateral
10	6	0.974	2017	Xingnao Kaiqiao	Unblocked collateral

The cluster analysis results mainly include Cluster ID, mean year, size, silhouette, label (LLR), and label (MI). Cluster ID is the number after clustering, and Size represents the number of members contained in the cluster. The larger the size is, the smaller the number. Mean Year represents the average year of the literature in the cluster, which can be used to judge the distance of the cited literature in the cluster. The larger the log-likelihood ratio (LLR) is, the more representative the cluster category; mutual information (MI) is mainly used to represent the relationship between terms and categories in text mining, and it does not consider the frequency of feature words.

### 3.7 Analysis of cited reference

**Figure 7:**
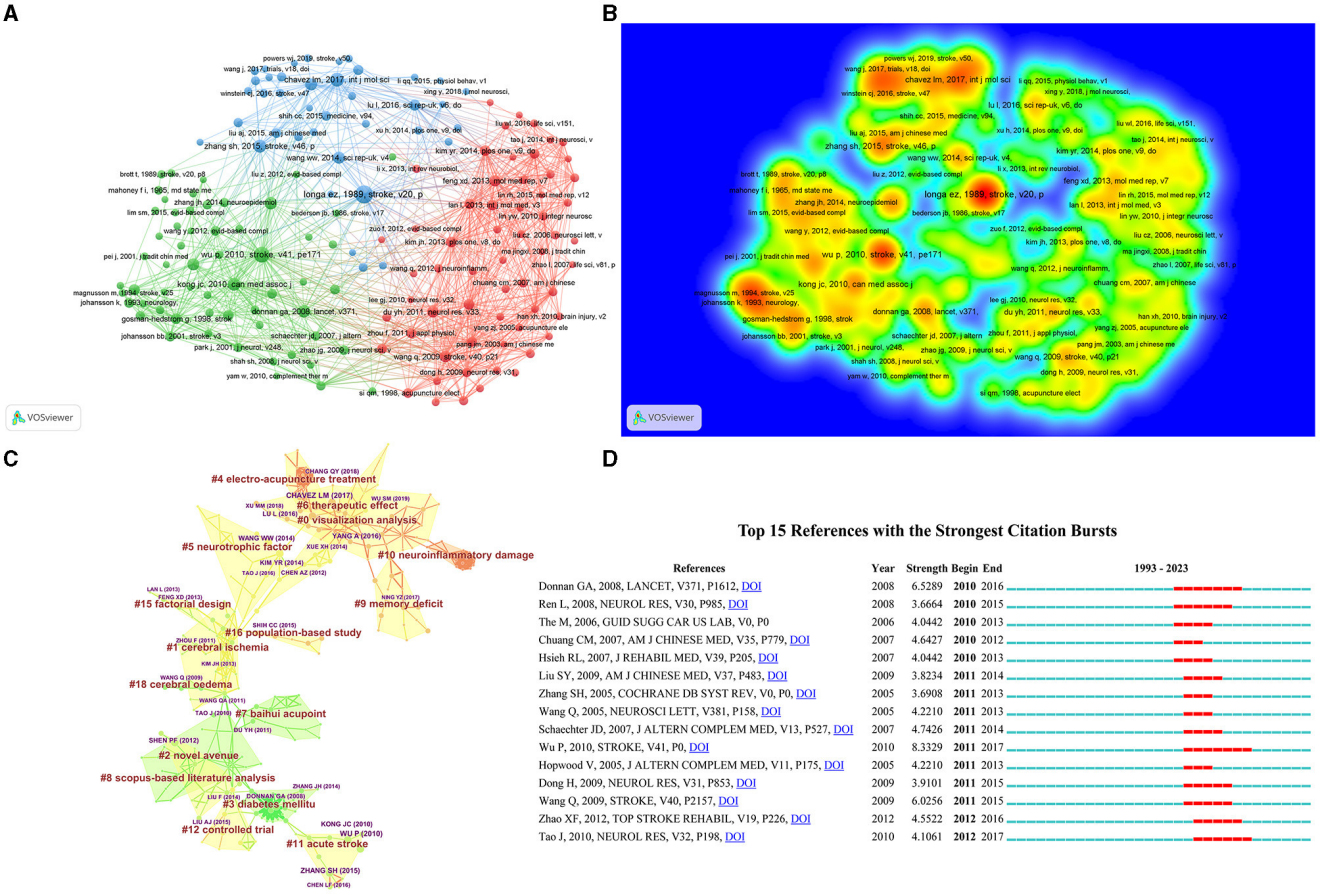
The visualization of cited reference **(A)**, Rainbow map **(B)**, Cluster map **(C)**, and Citation bursts **(D)** of cited reference based on label clusters with title terms. (**A**: different nodes represent different cited reference, and the size of the nodes represents the number of publications; **B**: the darker the yellow, the higher the frequency of appearance; **C**: different color blocks represent different clustering categories; **D**: the red bar represents frequent citations, while the green bar represents infrequent citations).

Cited references refer to the phenomenon in which two references are cited by the same reference. By analyzing the clustering and key nodes in the co-citation network, the knowledge structure of a certain research field can be revealed. The top 10 references in frequency and centrality are listed in Table 2. Among them, the top 5 references (Figures 7A, B) with the highest co-citations are Wu et al. (20) (Frequency = 41), Chavez et al. (22) (Frequency = 40), Zhang et al. (24) (Frequency = 36), Yang et al. (26) (Frequency = 30), Wang et al. (37) (Frequency = 27), and the highest centrality is Wang et al. (21) (Centrality = 0.58), which is regarded as the most noteworthy reference in the field, followed by Lin and Hsieh (23) (Centrality = 0.54), Lu et al. (25) (Centrality = 0.52), Kim et al. (27) (Centrality = 0.51), and Xie et al. (29) (Centrality = 0.48).

The co-citation map of references indicates the scientific relevance of the publications (Figure 7C), wherein the Modularity Q = 0.857 > 0.7, which enlightens the network, was rational. In the cluster atlas, the 15 largest clusters (Table 8) (small clusters were automatically filtered) included #0 visualization analysis, #1 cerebral ischemia, #2 novel avenue, #3 diabetes mellitu, #4 electro-acupuncture treatment, #5 neurotrophic factor, #6 therapeutic effect, #7 Baihui acupoint, #8 Scopus-based literature analysis, #9 memory deficit, #10 neuroinflammatory damage, #11 acute stroke, #12 controlled trial, #15 factorial design, and #16 population-based study. The clustering analysis results show that the research in this field involves not only the selection of acupuncture points but also the study of acute cerebral infarction and its complications, as well as randomized controlled studies on acupuncture for cerebral infarction. In our study, Burst History listed a total of 15 references with strong citation bursts. In Figure 7D, the red bar surrogates the outbreak of strong citations, of which the hot reference is Wu Ping's systematic evaluation study on the application of acupuncture in post-stroke rehabilitation published in Stroke Journal in 2010, which provides the possibility for acupuncture to treat the sequelae of cerebral infarction.

**Table 8:** Cited reference concerned with acupuncture for cerebral infarction that details of knowledge clusters.

**Cluster ID**	**Size**	**Silhouette**	**Mean (Year)**	**Label (LLR)**	**Label (MI)**
0	41	0.819	2018	Visualization analysis	Ischemic stroke rehabilitation
1	30	0.847	2013	Cerebral ischemia	Ischemic stroke rehabilitation
2	25	0.881	2008	Novel avenue	Ischemic stroke rehabilitation
3	21	0.931	2008	Diabetes mellitu	Ischemic stroke rehabilitation
4	18	0.969	2018	Electro-acupuncture treatment	Ischemic stroke rehabilitation
5	18	0.944	2014	Neurotrophic factor	Ischemic stroke rehabilitation
6	17	0.928	2016	Therapeutic effect	Ischemic stroke rehabilitation
7	16	0.945	2007	Baihui acupoint	Ischemic brain injury
8	16	0.959	2011	Scopus-based literature analysis	Ischemic stroke rehabilitation
9	15	0.960	2017	Memory deficit	Spastic paresis
10	14	0.989	2020	Neuroinflammatory damage	Ischemic stroke rehabilitation
11	12	0.939	2013	Acute stroke	White matter microstructure
12	11	0.925	2013	Controlled trial	Cerebral ischemia
15	7	0.926	2012	Factorial design	Cerebral ischemia
16	6	0.943	2014	Population-based study	Cerebral ischemia

The cluster analysis results mainly include cluster ID, mean year, size, silhouette, label (LLR), and label (MI). Cluster ID is the number after clustering, and Size represents the number of members contained in the cluster. The larger the size is, the smaller the number. Mean Year represents the average year of the literature in the cluster, which can be used to judge the distance of the cited literature in the cluster. The larger the log-likelihood ratio (LLR) is, the more representative the cluster category; mutual information (MI) is mainly used to represent the relationship between terms and categories in text mining, and it does not consider the frequency of feature words.

## 4 Discussion

According to the trend of annual publication output, this field has shown stable development in the past (1993–2007), then slow development (2008–2013), fluctuating development (2013–2017), and rapid growth in the later period (2018–2023). The changes in development speed may be attributed to the development history of acupuncture abroad and changes in Chinese domestic traditional Chinese medicine policy. Following the joint establishment of the “London College of Traditional Chinese Medicine” with the Beijing University of Chinese Medicine in 1993, the UK now has four universities offering undergraduate courses in traditional Chinese medicine and acupuncture. In 2001, he began to advocate the legislation of traditional Chinese medicine and acupuncture, and the Traditional Chinese Medicine Management Committee was established in 2004. By 2008, the legislative group of the British Ministry of Health submitted a legislative proposal for “acupuncture, herbal medicine, and traditional Chinese medicine” to the government (38). There are nearly 20 kinds of diseases treated by acupuncture, and expenses are reimbursable. In addition, acupuncture has been officially recognized by Switzerland, Austria, the Netherlands, Denmark, Belgium, Russia, and other countries. The “Traditional Chinese Medicine Law of the People's Republic of China” came into effect on July 1, 2017 (39). As the first comprehensive law that comprehensively and systematically reflects the characteristics of traditional Chinese medicine, it reflects the great importance that the Chinese government attaches to the cause of traditional Chinese medicine. The development of the industry is of milestone significance. As an important part of traditional Chinese medicine, acupuncture will naturally develop more rapidly with policy support. These patterns can also be further confirmed by the development trend chart of the cumulative number of publications.

The main countries in the research of acupuncture treatment for cerebral infarction are China, the USA, and South Korea. The countries with the greatest cooperation are China and the USA, and China and the UK. The areas with the most intensive cooperation are mainly concentrated among developed countries in Europe. Ninety percentage of the top 10 institutions are from China, with Tianjin University of Traditional Chinese Medicine, Beijing University of Chinese Medicine, Guangzhou University of Chinese Medicine, and Shanghai University of Traditional Chinese Medicine as the core regions for closer cooperation. Most of these collaborations are limited to higher education institutions in traditional Chinese medicine in China, and there is a serious lack of cooperation with foreign institutions. Therefore, we suggest that in-depth cooperation between institutions in various countries can be strengthened to promote rapid progress in the cerebral infarction treatment with acupuncture.

Among the top 10 journals with the most publications, Lancet has the highest impact factor of 168.9, making it the top journal in the field. “Stroke” has an impact factor of 8.4, making it the most prolific journal in this field along with “Evidence-based Complementary and Alternative Medicine” and “Plos One.” The most important journal is “Acupuncture Electro Therapeutics Research.” These journals can help us quickly find the main results in the field. In addition, through frequency and Centrality, we can see that most journals are mainly professional journals on traditional Chinese medicine, and there are fewer comprehensive journals. This is the direction of future development of the field. Cluster analysis of journals can reveal the main research content that journals in this field focus on, including risk factors (diabetes), electroacupuncture treatment effects, post-stroke rehabilitation, motor function recovery, etc.

Tao Jing stands as an prominent author in the field of acupuncture treatment for cerebral infarction. She is known for her long-term work in the basic and clinical research of integrated traditional Chinese Medicine and Western medicine rehabilitation and has the highest publication output in the research of acupuncture treatment for cerebral infarction. Chen Lidian is from the Fujian University of Traditional Chinese Medicine. He innovates the key rehabilitation technologies of traditional and Western medicine in combination with post-stroke dysfunction and establishes a new system suitable for the dysfunction of functional dysfunction in China. The research on the compatibility effect of the acupoint and its mechanism is the main research. Meng Zhihong belongs to the first affiliated hospital of Tianjin University of Traditional Chinese Medicine, and the treatment method is mainly based on the tunnel. They come from higher education institutions or university-affiliated hospitals in the field of Chinese medicine in China. This pattern can also be seen through the cited author. Cluster analysis shows that electroacupuncture mainly suppresses autophagy through the SIRT1-FOXO1 signaling pathway, thereby improving cerebral ischemia/reperfusion injury. Electroacupuncture improves the neural plasticity of the motor cortex and corticospinal tract after cerebral infarction. Mei et al. (40) further enhanced the expression of SIRT1 through pretreatment with Baihui, Quchi, and Zusanli, suppressed the levels of Ac-FOXO1 and Atg7, and effectively improved neurological deficits and stroke volume in rats. Significant increase in dendritic spine density and inhibition of autophagy in ischemic cortical neurons indicate that electroacupuncture plays a neuroprotective role during the reperfusion period after MCAO in rats. Zhang et al. (41) found that acupuncture at the Zusanli and Neiguan acupoints in p-MCAO rats promoted the neural plasticity of axonal regeneration activation in the contralateral cortex and corticospinal tract of the mTOR signaling pathway, promoting neurological function recovery. The prominent cited author is Si et al. (42). Research has shown that acupuncture at Baihui, Shenting, Hegu, Neiguan, Sanyinjiao, and Taichong can not only increase cerebral blood flow but also promote vasodilation, further activating thrombolysis or reducing intracranial pressure, to improve and alleviate ischemic brain injury.

The frequency analysis of keywords, such as “recovery,” “rehabilitation,” and “expression,” has a high frequency, indicating that the rehabilitation treatment of transient cerebral infarction is the main direction, which includes both randomized controlled clinical trials and basic research on related mechanisms. The main research content involved in keyword clustering analysis includes inhibiting NF kappa B mediated activation, MCP-induced protein, memory deficit, and Xingnao Kaiqiao. Liu et al. (43) studied that acupuncture at the Baihui and Dazhui acupoints of pMCAO-induced SD rats can reduce the expression of IBA-1 and CD11B, inhibit the expression of NF-κB P65, IL-1β, and TNF-α, reduce the small glue after ischemic glue the transformation of quality cells to the M1 phenotype, ultimately achieving the goal of alleviating the inflammatory response after ischemic stroke. Jin et al. (44) focuses on the effects of acupuncture at Baihui Point on wild-type and MCPIP1 knockout mice induced by MCAO-induced brain inflammation and ischemic brain injury. After acupuncture treatment, it reduces infarct volume, neurological deficits, upregulation of pro-inflammatory cytokines, and leukocyte infiltration in the mouse brain, thereby exerting neuroprotective effects on the brain. Liu et al. (45) observed the effect of acupuncture treatment on the expression of Ref-1 in the hippocampus of rats with multiple cerebral infarctions. The results showed that acupuncture increased the expression of CA1, CA3, and dentate gyrus in the hippocampus of rats, as well as enhanced SOD activity, to improve reference memory therapy for memory deficits. Cui and Jia (46) added Xingnao Kaiqiao acupuncture based on sodium butylphthalide treatment. The results displayed that this can significantly improve neurological deficits and cognitive impairment. The study by Song et al. (47) demonstrates that the acupuncture method of “awakening the brain and opening the mind” can not only interfere with the inflammatory response of the brain after cerebral ischemia-reperfusion but also improve cognitive function, which may play a role in the downregulation of serum MMP-2 and MMP-9 levels and the increase in IGF-1 levels. Burst detection analysis of keywords found that neural regeneration is the hot research topic in this field.

This exploration summarizes the Cited Reference of acupuncture for cerebral infarction: The important literature is Longa EZ's study (48), titled “Reversible middle cerebral artery occlusion without Craniotomy in rates,” published in the journal “Stroke” in 1989. The research induced infarction in the right middle cerebral artery (MCA) through *in vitro* vascular occlusion to establish this animal model, providing methodological guarantees for future scholars to study reversible local ischemia in rats without craniotomy. Prominent documents include Wu P's “Acquisition in Poststroke Rehabilitation: A Systematic Review and Meta-Analysis of Randomized Trials” study published in 2010 in the journal Stroke (20) and Donnan GA's “Stroke” article published in Lancet in 2008 (35). Wu et al. evaluated the rehabilitation application of acupuncture treatment after stroke through systematic evaluation and meta-analysis, confirming the therapeutic effect of acupuncture treatment for cerebral infarction. On the other hand, Donnan GA understood cerebral infarction from multiple levels, such as epidemiology, pathophysiology, classification, stroke prognosis, acute intervention, nursing, and prevention.

Cited Reference clustering research mainly includes the following parts: ① **Baihui acupoint:** Wang et al. (49) studied that acupuncture at Baihui acupoint activates rats through activation α 7nAChR inhibits the release of HMGB1 to protect the brain from transient ischemic injury. The discovery exhibits that it provides new treatment strategies for intervention after acupuncture for stroke. Wang Yaling's research (50) observed how electroacupuncture regulates Treg/γδ the balance of T cells can improve the inflammatory damage in the intracranial and intestinal tracts of rats with cerebral infarction. Acupuncture treatment not only reduces the volume of cerebral infarction in rats with cerebral infarction but also reduces the damage to the intestinal barrier. This provides us with new insights into the treatment of cerebral infarction from the perspective of the brain-gut axis. Guo et al. (51) explored the protective effect of acupuncture at Baihui point on cerebral ischemic injury in diabetes mice and further studied the role of NADPH oxidase-mediated oxidative stress. Acupuncture can not only reduce the infarct area but also lower the levels of MDA and ROS in the brain, and inhibit the activation of NADPH oxidase, thereby exerting a neuroprotective effect. These are important contents of research on acupuncture at Baihui Point for the treatment of cerebral infarction. ② **memory deficit:** This study mainly concentrates on the effect of acupuncture on memory impairment caused by multiple cerebral infarctions. The main research is conducted by Liu Cunzhi's team at Beijing University of Chinese Medicine. Acupuncture treatment for cerebral infarction can improve memory impairment by downregulating the expression of pro-apoptotic factor Bax in the hippocampal CA1 region, upregulating the expression of anti-apoptotic factor Bcl-2, and regulating the disordered proportion of Bcl-2 family genes to resist cell apoptosis (52). Furthermore, the author's research also found that acupuncture treatment significantly increased the expression of CuZnSOD mRNA and protein in the hippocampus of the injured rats, and then increased the activities of superoxide dismutase and glutathione peroxidase in the hippocampus to improve the oxidative damage after cerebral infarction (53). These studies have enriched the main content of the mechanism of acupuncture in treating memory impairment after cerebral infarction. ③ **Neurotrophic factor:** The neuroprotective mechanism of acupuncture at Baihui and Dazhui in the treatment of mild stroke is carried out by activating the MEK1/2/ERK1/2/p90RSK/bad signaling pathway mediated by brain-derived neurotrophic factor, further achieving the prevention of cell Apoptosis (54). Moreover, similar studies include: Acupuncture can improve the recovery of motor function after cerebral infarction (31). This mechanism is mainly related to the increased expression of BDNF related to motor recovery. It extends a strategy for the treatment of this disease and deserves further study. ④ **Controlled trial**: From the perspective of cerebral infarction staging, acupuncture treatment of this disease includes both the treatment of acute cerebral infarction (28) and the treatment of sequelae of cerebral infarction (hemiplegia after cerebral infarction, mild cognitive impairment after cerebral infarction) (55, 56). In terms of treatment methods, some methods combine acupuncture and rehabilitation training, as well as methods that combine acupuncture and medication, and a combination of scalp acupuncture and electromagnetic convergence stimulation therapy (57), all of which enrich the research content of acupuncture treatment for cerebral infarction. Furthermore, there are systematic reviews and meta-analyses of relevant randomized controlled trials based on evidence-based medicine (58). These studies show that the effect of acupuncture in treating cerebral infarction is unique and the effect is obvious. However, the limitation lies in the need for randomized controlled trials with a larger sample size will provide further insight into the therapeutic effects of inflammation. This is also the main direction of future randomized trial research. The published paper further demonstrates the clinical efficacy of acupuncture in treating ischemic stroke. Li et al. (10) observed the clinical effect of sciatic nerve acupuncture in treating lower limb hemiplegia after cerebral infarction through a randomized controlled trial and provided a new complementary and alternative therapy for the recovery of limb function after stroke through the design of clinical plans. Chen et al. (59) conducted a randomized controlled trial combined with neuroimaging to observe the therapeutic effect of acupuncture on post-stroke hemiplegia in Hegu, Neiguan, Quchi, Zusanli, Yanglingquan, and Sanyinjiao. The results showed that acupuncture can promote motor recovery and improve cerebellar VMHC in post-stroke hemiplegic patients through bilateral static and dynamic recombination. These new clinical studies further demonstrate the precise efficacy of acupuncture in treating complications and limb hemiplegia after cerebral infarction. ⑤ **Population-based study**: This clustering content mainly finds that acupuncture treatment can reduce the risk of stroke through retrospective cohort studies. Ton et al. (60) detected that acupuncture can diminish the risk of stroke after Bell's palsy. However, the limitation of this study is that the characteristics of the database limit the accuracy of the research results. Further validation is needed in large-scale prospective studies in the future. Shih et al. (61) analyzed through a cohort study that acupuncture can reduce the risk of stroke in patients with traumatic brain injury in Taiwan, China. However, due to the lack of information about lifestyle, biochemical characteristics, severity of traumatic brain injury, and acupoints used in treatment, this study was limited. These studies offer us valuable research ideas for conducting large-scale prospective studies.

## 5 Conclusions

This research offers a new perspective on the trends of acupuncture for cerebral infarction. Despite definite limitations, this study wholly declares the global trend of acupuncture for cerebral infarction and bespeaks it to readers in the shape of a visual mapping knowledge domain. Most papers in this field were published in China, the countries with the closest cooperation are mostly concentrated in Europe, and institutional cooperation is limited to higher education institutions in traditional Chinese medicine in China. The author who has published the most relevant papers is Tao Jing, the most cited author is Zhang SH, the prominent cited author is Si QM. The journal that has published the most papers in this field is Stroke, and the most key journal is Acquisition Electric Therapeutics Research, the hotspot of the keyword is nerve regeneration, and noteworthy reference is Wu et al. (20). Furthermore, future research can focus on large-scale randomized controlled trials and cohort studies to further verify its clinical treatment effects. Moreover, acupuncture rehabilitation treatment for complications and sequelae of cerebral infarction is the main direction. The hotspot of acupuncture for ischemic stroke mainly focuses on nerve regeneration. Acupuncture can improve the proliferation and differentiation of nerve cells into mature neurons, which may be one of the key reasons why acupuncture can improve neurological dysfunction, providing new ideas for scholars engaged in this field in the future. In brief, the results of this research may bring researchers useful information, such as research frontiers, potential collaborators, Countries, cooperative institutions, preponderant research content, and hotspots in the field.

## 6 Research limitations

There are several limitations of this study. Firstly, although the search strategy searches for synonyms of the MeSH subject words in PubMed, it may still cause some works of literature to be missed. Secondly, in our research, we only use the Science Citation Index-Expanded (SCIE) module in the Web of Science database. Although it contains most of the literature needed for research, it may still cause the loss of some literature.

## Data availability statement

The original contributions presented in the study are included in the article/Supplementary material, further inquiries can be directed to the corresponding authors.

## Author contributions

YZ: Writing—original draft, Writing—review & editing. LH: Data curation, Methodology, Writing—review & editing. WL: Funding acquisition, Resources, Validation, Visualization, Writing—original draft. LC: Writing—review & editing, Validation, Funding acquisition.
