# Supplementary material for: A bibliometric analysis of acupuncture for cerebral infarction from 1993 to 2023
Source: Front Neurol. 2024 May 2;15:1386164. doi: 10.3389/fneur.2024.1386164 (PMC11096454; doi:10.3389/fneur.2024.1386164)
Supplement: Supplementary file 1 [file Table_1.pdf]

Table S1 Retrieval Strategy of acupuncture for Cerebral Infarction

No.	Search strategy	Results
1	TS= “cerebral infarction” AND “acupuncture”	147
2	TS= “cerebral infarction” AND “acupuncture therapy”	54
3	TS= “cerebral infarction” AND “Acupuncture, Ear”	0
4	TS= “cerebral infarction” AND “Acupuncture Points”	13
5	TS= “cerebral infarction” AND “Acupuncture Analgesia”	0
6	TS= “ischemic stroke” AND “acupuncture”	374
7	TS= “ischemic stroke” AND “acupuncture therapy”	155
8	TS= “ischemic stroke” AND “Acupuncture, Ear”	0
9	TS= “ischemic stroke” AND “Acupuncture Points”	52
10	TS= “ischemic stroke” AND “Acupuncture Analgesia”	2
